# Infectious events in patients with severe COVID-19: results of a cohort of patients with high prevalence of underlying immune defect

**DOI:** 10.1186/s13613-021-00873-x

**Published:** 2021-05-25

**Authors:** Anastasia Saade, Giulia Moratelli, Guillaume Dumas, Asma Mabrouki, Jean-Jacques Tudesq, Lara Zafrani, Elie Azoulay, Michael Darmon

**Affiliations:** 1 Service de médecine Intensive et de réanimation, hôpital Saint-Louis, 1 Avenue Claude Vellefaux, 75010 Paris, France; 2 Université de Paris, ECSTRA Team, UMR 1153, Center of Epidemiology and Biostatistics, INSERM, Paris, France

**Keywords:** COVID-19, SARS-CoV-2, Community-acquired infections, Hospital-acquired infections, Pneumonia, Intensive care unit

## Abstract

**Background:**

Empirical antibiotic has been considered in severe COVID-19 although little data are available regarding concomitant infections. This study aims to assess the frequency of infections, community and hospital-acquired infections, and risk factors for infections and mortality during severe COVID-19.

**Methods:**

Retrospective single-center study including consecutive patients admitted to the intensive care unit (ICU) for severe COVID-19. Competing-risk analyses were used to assess cumulative risk of infections. Time-dependent Cox and fine and gray models were used to assess risk factors for infections and mortality. Propensity score matching was performed to estimate the effect of dexamethasone.

**Results:**

We included 100 patients including 34 patients with underlying malignancies or organ transplantation. First infectious event was bacterial for 35 patients, and fungal for one. Cumulative incidence of infectious events was 27% [18–35] at 10 ICU-days. Prevalence of community-acquired infections was 7% [2.8–13.9]. Incidence density of hospital-acquired infections was 125 [91–200] events per 1000 ICU-days. Risk factors independently associated with hospital-acquired infections included MV. Patient’s severity and underlying malignancy were associated with mortality. Dexamethasone was associated with increased infections (36% [20–53] vs. 12% [4–20] cumulative incidence at day-10; *p* = 0.01). After matching, dexamethasone was associated with hospital-acquired infections (35% [18–52] vs. 13% [1–25] at 10 days, respectively, *p* = 0.03), except in the subset of patients requiring MV, and had no influence on mortality.

**Conclusions:**

In this population of COVID-19 patients with high prevalence of underlying immune defect, a high risk of infections was noted. MV and use of steroids were independently associated with infection rate.

**Supplementary Information:**

The online version contains supplementary material available at 10.1186/s13613-021-00873-x.

## Introduction

Recently, the SARS-CoV-2 outbreak, first detected in Wuhan, China, on December 31 2019, has been responsible for a quickly spreading pandemic [1–5].

About one in five patients requires intensive care unit (ICU) admission, in most cases for severe acute respiratory syndrome [6], although shock and acute kidney injury are common [7, 8].

Due to the suspicion of concomitant community-acquired infections, and expected increase in hospital-acquired infection (hospital-acquired infections) secondary to immune defects, including lymphopenia and COVID-19-induced immunoparalysis [9, 10], empiric antibiotic therapy has been clinically considered in critically ill COVID-19 patients [11]. However, data regarding the risk of infectious events in patients with SARS-CoV-2 infection are scarce. Recently, studies have suggested a rate of associated bacterial infections up to 25% [2, 3, 12–14]. However, more insights into the risks for hospital-acquired infections and their impact on morbidity and mortality are warranted. For instance, lack of distinction between community-acquired infections and hospital-acquired infections and failure to take into account the influence of competing factors and time-dependent competing events hamper conclusions to be driven by the existing literature. Moreover, data to challenge existing guidelines in the sickest patients may be warranted.

This study aims to assess the frequency of infectious events during the course of severe COVID-19 and risk factors for infections and mortality.

Primary objective was to assess the incidence of infectious events in SARS-CoV-2 patients admitted to the ICU. Secondary objectives were to assess prevalence of community-acquired infections and incidence density of hospital-acquired infections, and to describe risk factors for infectious events and for mortality at 28 days during the course of severe COVID-19 before and after propensity score matching to estimate the effect of dexamethasone.

## Methods

### Study design

This study was approved by an institutional review board (French Intensive Care Society—CE SRLF no. 20-32). Need for informed consent was waived with regard to the study observational design and in accordance with the French law. This study was conducted in accordance with the principles of the Declaration of Helsinki.

We performed a retrospective single-center study in the ICU of Saint-Louis University Hospital, Paris, France. Consecutive adult patients admitted in our ICU for COVID-19 between March 10 and May 10 2020 were included.

Admission to the ICU from the wards was at the physician’s discretion and according to bed availability. Briefly, COVID-patients receiving oxygen were evaluated, most of the patients being admitted for respiratory failure when oxygen requirement was > 6 L/min.

Patients admitted for COVID-19 received empiric antibiotic therapy including third generation cephalosporin and spiramycin, which was stopped after bacterial infection was ruled out.

Except for non-invasive mechanical ventilation and high flow nasal oxygen (which were partly avoided during the first 4 weeks of the first wave), organ support was provided in accordance with usual guidelines.

Decision to add dexamethasone to the standard of care was done after multidisciplinary meeting on clinical basis in patients whose respiratory status was worsening or failed to improve. It was based on a thorough consideration of the following elements: ICU admission > 7 days from onset and absence of clinical evidence of bacterial infection. Dexamethasone was used at 20 mg per day for 5 days followed by 10 mg per day for 5 days. No other steroid was used in this study.

Biological sampling was systematically performed at ICU admission for all patients as part of the routine management of COVID-19 patients [15], and included blood gas with lactate, blood count, renal and hepatic assessment, urinary assessment, hemostasis, electrophoresis of serum proteins, lymphotyping, interleukin-6 (IL-6) dosage, peripheral and catheter blood cultures, cytobacteriological examination of the urine, nasopharyngeal swab RespiFinder^®^ SMART-22 FAST assay (Pathofinder), cytobacteriological examination of the sputum, tracheal suction or protected distal swab in case of intubation, galactomannan antigenemia, β-d-glucan, CMV PCR, EBV PCR and HIV screening.

Infectious screening including peripheral and catheter blood cultures, cytobacteriological examination of the urine, nasopharyngeal swab RespiFinder^®^ SMART-22 FAST assay (Pathofinder), cytobacteriological examination of the sputum, tracheal suction or protected distal swab in case of intubation were performed in case of clinical suspicion of sepsis.

Galactomannan antigenemia, β-d-glucan, CMV PCR, EBV PCR were performed at ICU admission twice a week, and repeated in case of sepsis during ICU stay.

### Data

Data were collected from patients’ charts, electronic records, and laboratory data on a case report form (CRF). Retrieved data included baseline demographic characteristics [age at admission, sex, body mass index (BMI)], comorbidities, onset of symptoms, hospital admission and ICU admission, symptoms at ICU admission, treatments prior to admission (NSAIDs, steroids, antibiotic therapy) and during ICU stay, comorbidities (pulmonary disease, smoking, cardiac disease, diabetes, blood hypertension, solid tumor, hematological malignancy, solid organ transplantation), intake of angiotensin-receptor blockers (ARBs), biological results and radiological findings at admission, date of positive SARS-CoV-2 PCR, vasopressor implementation, intubation, prone position, treatment with dexamethasone, treatment with immunotherapies, date of concomitant infection, microbiological documentation, antibiotic regimens, and ICU mortality or discharge to the wards.

Severity was assessed using Simplified Acute Physiology Score (SAPSII) during first 24 h [16].

Dataset was check for missing data, internal and external validity, and potential discrepancies were audited before statistical analysis.

### Clinical definitions

Severe COVID-19 patients were defined as patients with COVID-19 requiring ICU admission.

Infections were defined as any clinical context suggesting fungal or bacterial infection with microbiological documentation of pathogenic microorganisms, and concomitant decision to initiate or adapt the antimicrobial therapy.

Community-acquired infections were defined as documented infections concurrent with the diagnosis of COVID-19, detected at hospital admission or within the first 48 h.

Hospital-acquired infections were defined as documented infections diagnosed after 48 h of hospital admission. Early hospital-acquired infections occurred between 48 h and 7 days of hospital stay while late hospital-acquired infections, occurred after 7 days.

The following infectious events were collected:

Lower respiratory tract infections were defined when a pathogenic respiratory germ was identified above the significant threshold on a respiratory sample (sputum assessment [≥ 10^7^ UFC/mL], tracheal suction [≥ 10^5^ UFC/mL] or protected distal catheter culture [≥ 10^4^ UFC/mL]) or on the respiratory panel BioFire^®^ FilmArray^®^ pneumonia plus panel [≥ 10^5^ UFC/mL] (BioFire Diagnostics, Biomerieux) in the presence of acute respiratory failure, clinical worsening and compatible clinical or radiological setting.

Ventilator-associated pneumonia (VAP) was defined upon 48 h of mechanical ventilation in case of new, persistent (> 48 h) or progressive radiographic infiltrate with at least two of the following: temperature of > 38 °C or < 36 °C, blood leukocyte count of > 10 G/L or < 5 G/L, purulent tracheal secretions, and gas exchange degradation; and the identification of a pathogenic respiratory germ on a respiratory sample (tracheal suction [≥ 10^5^ UFC/mL], protected distal catheter [≥ 10^4^ UFC/mL], bronchoalveolar lavage culture [≥ 10^4^ UFC/mL]) or on the respiratory panel BioFire^®^ FilmArray^®^ pneumonia plus panel [≥ 10^5^ UFC/mL] (BioFire Diagnostics, Biomerieux).

Bacteremia was defined when a pathogenic germ was detected on a single blood culture or, in case of blood culture contaminants (e.g., coagulase-negative Staphylococcus), significance was considered when at least two blood cultures were positive with similar phenotypes, and with clinical relevance [17].

Fungal infection was defined when a fungemia by any *Candida sp.* was detected. Identification of Candida species on respiratory samples were considered as respiratory tract colonization [18].

*Aspergillus fumigatus* infection was defined by the detection of *Aspergillus fumigatus* in respiratory tract samples when infection was considered probable or proven according to the European Organization for Research and Treatment of Cancer Mycoses Study Group (EORTC-MSG) consensus criteria in immunocompromised patients [19].

### Statistical analysis

We calculated, a priori, that 100 patients would allow to identify a risk factor of infections resulting in a risk odds ratio (OR) of 1.25, assuming 40% incidence of infections with 90% statistical power and an alpha risk of 0.05.

Data are reported as absolute value with percentage or median with interquartile interval.

No adjustment for multiple comparisons was performed in this study. To avoid false positive results, bivariate results were planned to be reported as exploratory and in way to understand variable selection process. Adjusted analyses and raw cumulative incidence were results of interest. Three preplanned adjusted analyses and a post hoc analysis were performed. This later is clearly identified through the manuscript.

Infections are reported as cumulative incidence, prevalence, or as incidence density for 1000 ICU-days. VAP are reported as incidence density for 1000 h of mechanical ventilation.

As the first infectious event is likely to influence the rate of subsequent infections, only first infectious events were analyzed.

A competing risk analysis was performed to assess the cumulative risk of infections. Concomitant competing risks were discharged alive from the ICU and ICU mortality.

Time-dependent Cox model and Fine and Gray model were used to assess risk factors of hospital-acquired infections and of day-28 mortality. Risk factors independently associated with community-acquired infections were not assessed due to the low number of events. Models were built using conditional backward stepwise variable selection process based upon variable influence in univariate analysis. Critical entry and exit *p* values were 0.2 and 0.1, respectively. Preplanned clinically relevant variables (preexisting immune defect, lymphopenia, SAPSII) were forced into the final model, if not previously selected.

Last, we performed a post hoc propensity score matching analyses to estimate the effect of dexamethasone on mortality. Briefly, the risk of receiving dexamethasone was assessed using logistic regression. A propensity score matching was performed without replacement on a 1:1 fashion and according to the closest neighbor method. Covariates that predicted receiving dexamethasone (critical entry and exit *p* values of 0.2 and 0.1, respectively) were cardiac diseases, onset of symptoms to admission, and treatment with eculizumab. Quality of matching was assessed using propensity score distribution and standardized mean difference of variables of interest before and after matching.

Influence of dexamethasone on mortality was assessed using Kaplan–Meier survival curve and log rank analysis. The risk of hospital-acquired infections using competing risk analysis was assessed before and after stratification for mechanical ventilation, which remained different across the groups.

Statistical significance was considered using two-sided tests with a critical alpha risk of 0.05.

Statistical analyses were performed using R version 3.4.4 (R Foundation for Statistical Computing).

## Results

**Fig. 1:**
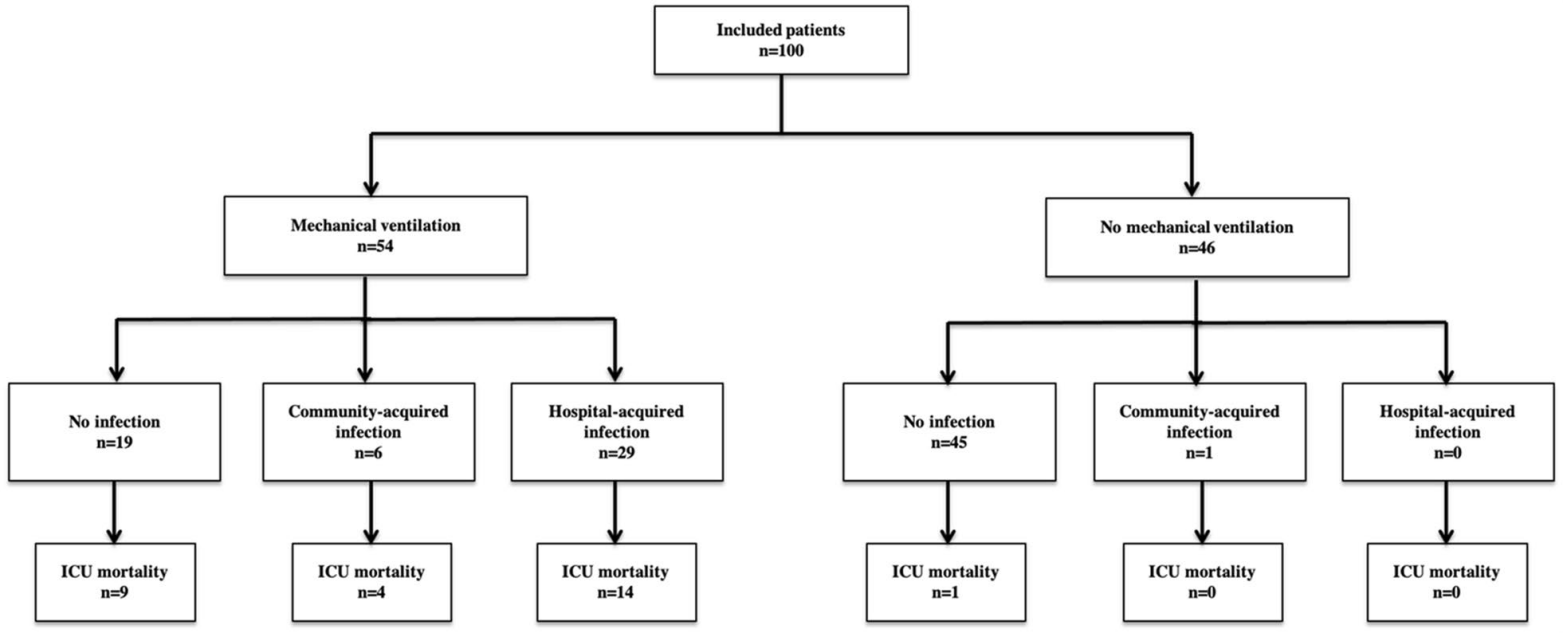
Study flow-chart

One hundred patients admitted to the ICU for a SARS-CoV-2 infection were included in the study (Fig. 1). Median age was 59 [53–67] years and most patients were male (73%). Median BMI was of 28 kg/m^2^ [24–31]. Most frequent comorbidities were blood hypertension (50%) and diabetes (27%). One-fourth of the patients (24%) had an underlying malignancy and 10% were solid organ transplant recipients.

Time between the onset of symptoms and ICU admission was 8 days [5–12]. Forty-five patients (45%) had received an antibiotic therapy and 19 steroids before ICU admission. Of the 19 patients, all of them had systemic steroids for underlying disease including low-dose steroids (< 0.25 mg/kg/day) for preexisting solid organ transplantation (*n* = 9), and remaining patients had underlying malignancies and intermittent high-dose steroids (0.25–1 mg/kg/day) (*n* = 10). None of the patients received dexamethasone as response to COVID-19 infection, before ICU admission.

At admission, median severity according to the SAPSII was 26 [18–39]. All patients exhibited lymphopenia and high IL-6 levels (Table 1). Fifty-four (54%) patients required mechanical ventilation and 48 (48%) vasopressors. Median ICU length of stay was 6 days [3–13] and ICU mortality reached 28% (*n* = 28) (Additional file 1: Table S1).

**Table 1:** Demographic and clinical characteristics of the study population

Type of infection	Community-acquired (*n* = 7)	Early hospital-acquired (*n* = 14)	Late hospital-acquired (*n* = 15)	No infection (*n* = 64)	*p*
Age (years)	58 [53–69]	58 [52–67]	64 [56–70]	58 [53–65]	0.589
Male	5 (71)	9 (64)	10 (67)	49 (77)	0.737
Comorbidities					
BMI (kg/m^2^)	31 [27–33]	28 [26–31]	29 [25–32]	27 [24–30]	0.550
Cardiac disease	3 (43)	3 (21)	2 (13)	10 (16)	0.320
COPD	0 (0)	1 (7)	0 (0)	1 (2)	0.494
Diabetes	3 (43)	6 (43)	3 (20)	15 (23)	0.325
Chronic kidney disease	1 (14)	2 (14)	2 (13)	9 (14)	1.000
Hypertension	5 (71)	7 (50)	9 (60)	29 (45)	0.485
Solid tumor	0 (0)	0 (0)	0 (0)	9 (14)	0.135
Hematological malignancy	3 (43)	2 (14)	2 (13)	8 (13)	0.202
SOT	1 (14)	2 (14)	1 (7)	6 (9)	0.887
Any ARBs	3 (50)	2 (14)	7 (47)	15 (23)	0.030
Delay since onset (days)	14 [11–17]	8 [5–10]	9 [6–12]	8 [5–11]	0.077
Exposure before admission					
NSAIDs	0 (0)	0 (0)	0 (0)	1 (2)	0.904
Corticosteroids	1 (14)	2 (14)	4 (27)	12 (19)	0.831
Antibiotics	2 (29)	7 (50)	6 (40)	30 (47)	0.766
ICU admission					
SAPSII	40 [28–42]	33 [24–47]	34 [24–50]	23 [18–33]	0.050
O_2_ (L/min)	4 [2–11]	11 [3–14]	9 [6–15]	8 [6–12]	0.479
Focal opacity at chest X-ray	1 (14)	0 (0)	2 (13)	2 (3)	0.197
PaO_2_/FiO_2_	147 [114–275]	113 [100–159]	156 [121–270]	157 [115–234]	0.166
Leukocytes (G/L)	7.3 [4.9–9.3]	6.9 [6.1–9.6]	8.3 [5.0–10.3]	7.1 [5.2–9.1]	0.614
Lymphocytes (G/L)	0.64 [0.46–0.93]	0.83 [0.77–1.48]	0.60 [0.38–0.98]	0.78 [0.55–1.14]	0.212
Lactate (mmol/L)	0.90 [0.80–1.60]	1.2 [1.0–1.4]	1.2 [1.1–1.6]	1.2 [0.9–1.5]	0.745
CPK (UI/L)	410 [155–579]	126 [119–328]	114.00 [63–346]	182 [80–372]	0.788
Creatinine (µmol/L)	65 [61–75]	89 [60–144]	70 [59–125]	80 [66–120]	0.591
IL-6 (ng/mL)	95 [70–121]	87 [47–291]	121 [83–186]	81 [40–111]	0.264
Gammaglobulin (g/L)	3 [3–11]	8 [8–12]	8 [7–11]	10 [8–12]	0.236
β-d-Glucan (pg/mL)	0 [0–0]	0 [0–0]	0 [0–177]	0 [0–0]	0.073
Therapeutics					
Dexamethasone	3 (50)	9 (64)	8 (53)	13 (20)	0.002
Eculizumab	1 (14)	0 (0)	3 (20.0)	6 (9)	0.335
Tocilizumab	0 (0)	0 (0)	0 (0)	5 (8)	0.398
Mechanical ventilation	6 (86)	14 (100)	15 (100)	19 (30)	< 0.001
Vasopressors	4 (67)	13 (93)	14 (93)	17 (27)	< 0.001
Renal replacement therapy	1 (17)	3 (21)	6 (40)	2 (3)	0.001
VAP (/1000 h of MV)	–	6.9[1.5–13.0]	4.4[2.8–6.7]	–	< 0.001
ICU Stay (days)	12[9–13]	17[13–25]	20[13–31]	4[2–6]	< 0.001
ICU mortality	4 (57)	8 (57)	6 (40)	10 (16)	0.002

Data are reported as absolute value with percentage for categorical variables or median with interquartile interval for quantitative variables. β-d-Glucan was considered positive when ≥ 80 pg/mL

*ARBs* angiotensin receptor blockers, *BMI* body mass index, *COPD* chronic obstructive pulmonary disease, *CPK* creatine phosphokinase, *ICU* intensive care unit, *MV* mechanical ventilation, *NSAIDs* non-steroid anti-inflammatory, *SAPSII* Simplified Acute Physiology Score II, *SOT* solid organ transplantation, *VAP* ventilator-associated pneumonia

Before ICU admission, hospital length of stay was 1 day [IQR 0–2] and patients received a median of 1 day [IQR 0–1] of antibiotics.

### Infectious events in severe SARS-CoV-2 patients

#### Overall rate of infection

Thirty-six patients had an infectious event during ICU stay. First infectious event was bacterial for 35 patients and fungal for one and included 7 community-acquired infections and 29 hospital-acquired infections (Table 1). Four patients developed a fungal infection after a bacterial event (Table 2).

**Table 2:** Microbial documentation of infectious events in ICU patients with severe COVID-19

Microbiological documentation	Community-acquired infections	Early hospital-acquired infections	Late hospital-acquired infections
Bacterial pneumonia			
* Acinetobacter baumannii*, n (%)	0 (0)	1 (3)	0 (0)
* Citrobacter koseri*,* n *(%)	0 (0)	1 (3)	1 (3)
* Escherichia coli*,* n *(%)	0 (0)	0 (0)	1 (3)
* Haemophilus influenzae*,* n *(%)	1 (3)	4 (11)	0 (0)
* Klebsiella sp.*,* n *(%)	1 (3)	3 (8)	1 (3)
* Moraxella catarrhalis*,* n *(%)	0 (0)	1 (3)	0 (0)
* Morganella morganii*,* n *(%)	0 (0)	0 (0)	1 (3)
* Pseudomonas aeruginosa*,* n *(%)	1 (3)	2 (6)	5 (14)
* Serratia sp.*,* n *(%)	1 (3)	0 (0)	1 (3)
* Staphylococcus aureus*,* n *(%)	1 (3)	2 (6)	4 (11)
* Stenotrophomonas maltophila*,* n *(%)	1 (3)	0 (0)	1 (3)
* Streptococcus constellatus*,* n *(%)	0 (0)	0 (0)	1 (3)
Bacteremia			
* Corynebacterium sp.*,* n *(%)	1 (3)	0 (0)	0 (0)
* Enterococcus faecalis*,* n *(%)	0 (0)	0 (0)	1 (3)
* Klebsiella pneumoniae*,* n *(%)	0 (0)	0 (0)	1 (3)
* Pseudomonas aeruginosa*,* n *(%)	0 (0)	0 (0)	1 (3)
* Streptococcus mitis*,* n *(%)	0 (0)	0 (0)	1 (3)
Fungal pneumonia^a^			
* Aspergillus fumigatus*,* n *(%)	1 (3)	0 (0)	2 (6)
Fungemia^a^			
* Candida albicans*,* n *(%)	0 (0)	0 (0)	2 (6)

^a^All fungal events are reported in the table, while only first bacterial events are given in the table

**Fig. 2:**
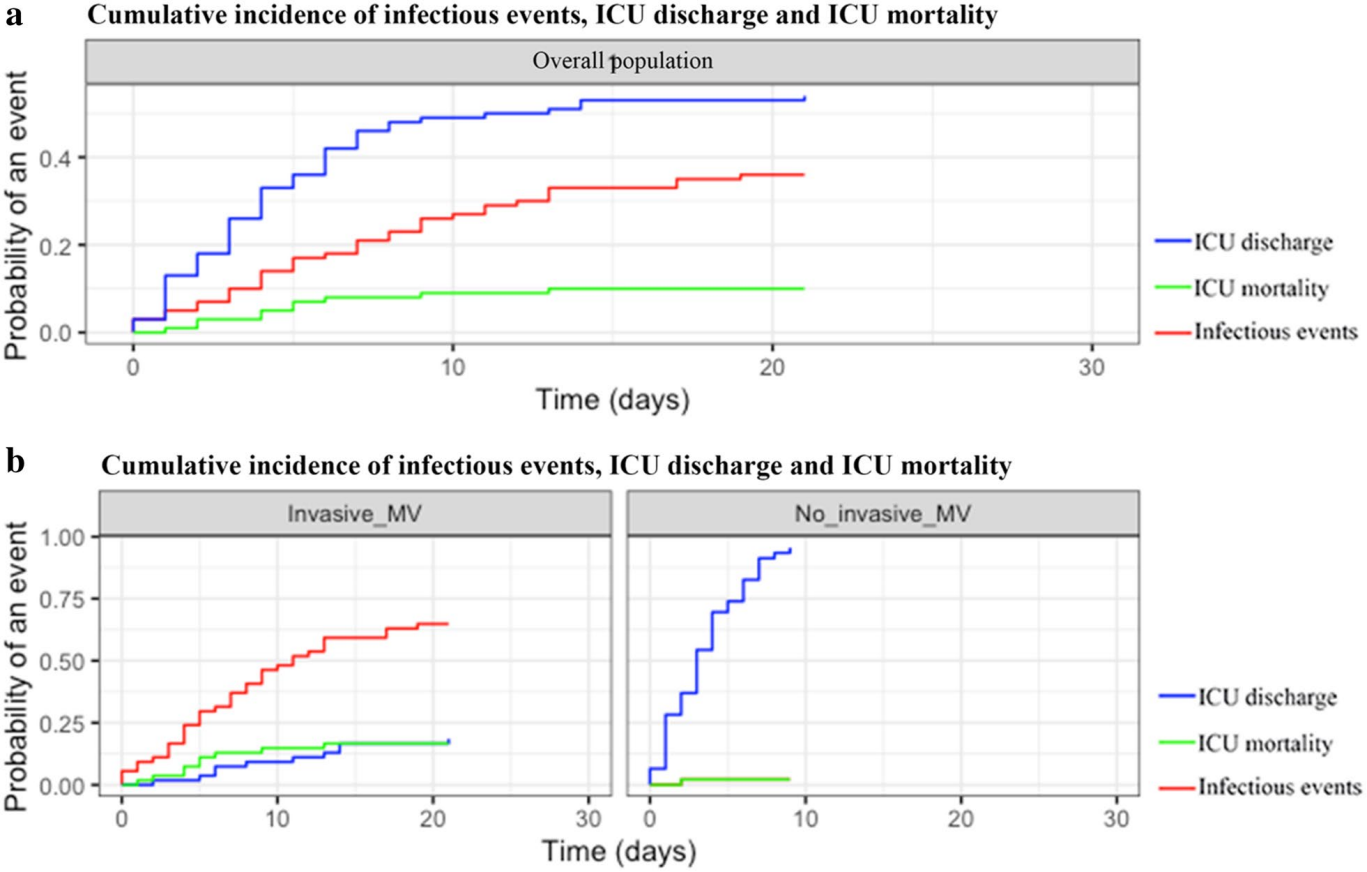
Competing risk of cumulative incidence of infectious events in patients admitted to the ICU for severe COVID-19. **a** Competing risk of cumulative incidence of infections (red), ICU mortality (green) and ICU discharge (blue) in overall patients. **b** Competing risk of cumulative incidence of infections (red), ICU mortality (green) and ICU discharge (blue) in patients according to mechanical ventilation

Overall proportion of patients developing infectious events after adjustment for time-dependent competing risk was of 27% [18–35] at 10 days. Cumulative incidence of infectious events in patients requiring mechanical ventilation was 48% [35–61] at 10 days while cumulative incidence was of 2.2% [0–6.5] at 10 days in patients without mechanical ventilation (*p* < 0.0001; Fig. 2).

#### Community-acquired infection

Prevalence of community-acquired infections was of 7% [2.8–13.9]. Main characteristics associated with community-acquired infections are reported in Table 1. Before ICU admission, only one and two patients had received, respectively, steroids and antibiotics without significant effect on the incidence of community-acquired infections (Table 1).

Enterobacteriaceae were frequently involved in community-acquired infections (Table 2). No influenza and influenza-like viruses (VRS or PIV) was detected.

### Hospital-acquired infections

**Fig. 3:**
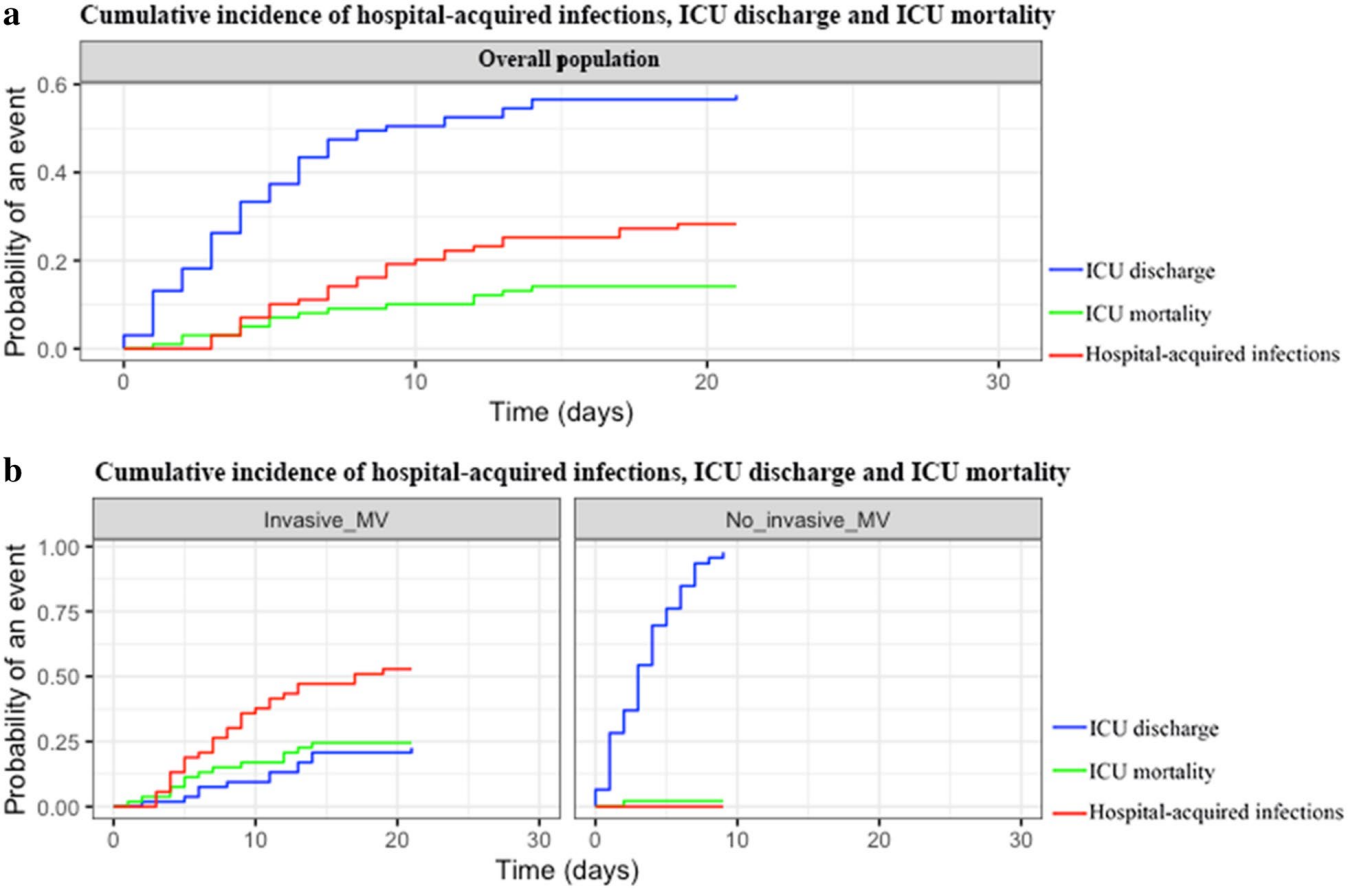
Competing risk of cumulative incidence of hospital-acquired infections in patients admitted to the ICU for severe COVID-19. **a** Competing risk of cumulative incidence of hospital-acquired infections (red), ICU mortality (green) and ICU discharge (blue) in overall patients. **b** Competing risk of cumulative incidence of hospital-acquired infections (red), ICU mortality (green) and ICU discharge (blue) in patients according to mechanical ventilation

Overall cumulative incidence of hospital-acquired infections was 20% [10, 12–27] at 10 days. Hospital-acquired infections occurred only in patients requiring mechanical ventilation (cumulative incidence of 38% [24–51] at 10 days vs*.* no hospital-acquired infections; *p* < 0.0001; Fig. 3).

Incidence density of hospital-acquired infections was 125 [91–200] events per 1000 ICU days and ranged between 225 [175–250] and of 91 [68–111] events per 1000 ICU days for early and late infections, respectively. The incidence density of VAP was 6 [2–8] events per 1000 h of mechanical ventilation. VAP occurred in a delay of 6 [5–9] days. Four patients with community-acquired infection secondarily developed a VAP during ICU stay (Table 1).

Mechanical ventilation, renal replacement therapy, vasopressors and the use of dexamethasone were associated with the occurrence of hospital-acquired infections (Table 1). Six patients had received steroids and 13 antibiotics before ICU admission (Table 1).

Main responsible pathogens are reported according to the delay of onset to hospital admission, in Table 2.

### Risk factors for hospital-acquired infection and mortality in severe COVID-19

Risk factors of hospital-acquired infections and ICU mortality are reported in Table 3.

**Table 3:** Covariates independently associated with infectious events, hospital-acquired infections and mortality in ICU patients admitted for severe COVID-19

Infectious events	Adjusted sHR	95% CI
BMI (kg/m^2^)	1.05	[0.99–1.108]
Mechanical ventilation	10.99	[1.48–82.04]
Hospital-acquired infections		
Leukocytes (G/L)	1.07	[1.01–1.14]
Vasopressors	4.44	[1.02–19.28]
Dexamethasone	1.77	[0.83–3.78]
Mortality		
Solid tumor	2.73	[1.01–7.42]
Hematological malignancy	1.99	[0.97–4.10]
SAPSII	1.04	[1.02–1.06]

*BMI* body mass index, *CI* confidence interval, *SAPSII* Simplified Acute Physiology Score II, *sHR* sub-hazard ratio

### Risk factors for hospital-acquired infections

After adjustment for confounders, mechanical ventilation remained the prominent factor independently associated with hospital-acquired infections (sHR 11.0; 95% CI [1.5–82.0]). Leukocytosis (sHR 1.1; 95% [1.0–1.1]) and vasopressor administration (sHR 4.4; 95% [1.0–19.3]) were also significantly associated with hospital-acquired infections. Dexamethasone was no longer associated with the occurrence of hospital-acquired infections (sHR 1.8; 95% [0.8–3.8]).

### Risk factors for mortality at 28 days

Main factors associated with ICU mortality were older age, cardiac comorbidities and severity at ICU admission. Occurrence of infectious events (64% vs. 26%) and VAP (46% vs. 19%) were also associated with ICU mortality (Additional file 1: Table S2).

After adjustment for confounders, underlying malignancy (HR 2.7; 95% CI [1.0–7.4]), and SAPSII (HR per SAPSII point 1.0; 95% CI [1.0–1.1]) were independently associated with mortality (Table 3). When forced in the final model, neither infections (HR 1.9; 95% CI [0.7–5.1]) nor dexamethasone (HR 0.6; 95% CI [0.1–3.1]), influenced the outcome.

### Use of dexamethasone and hospital-acquired infections in severe COVID-19

**Fig. 4:**
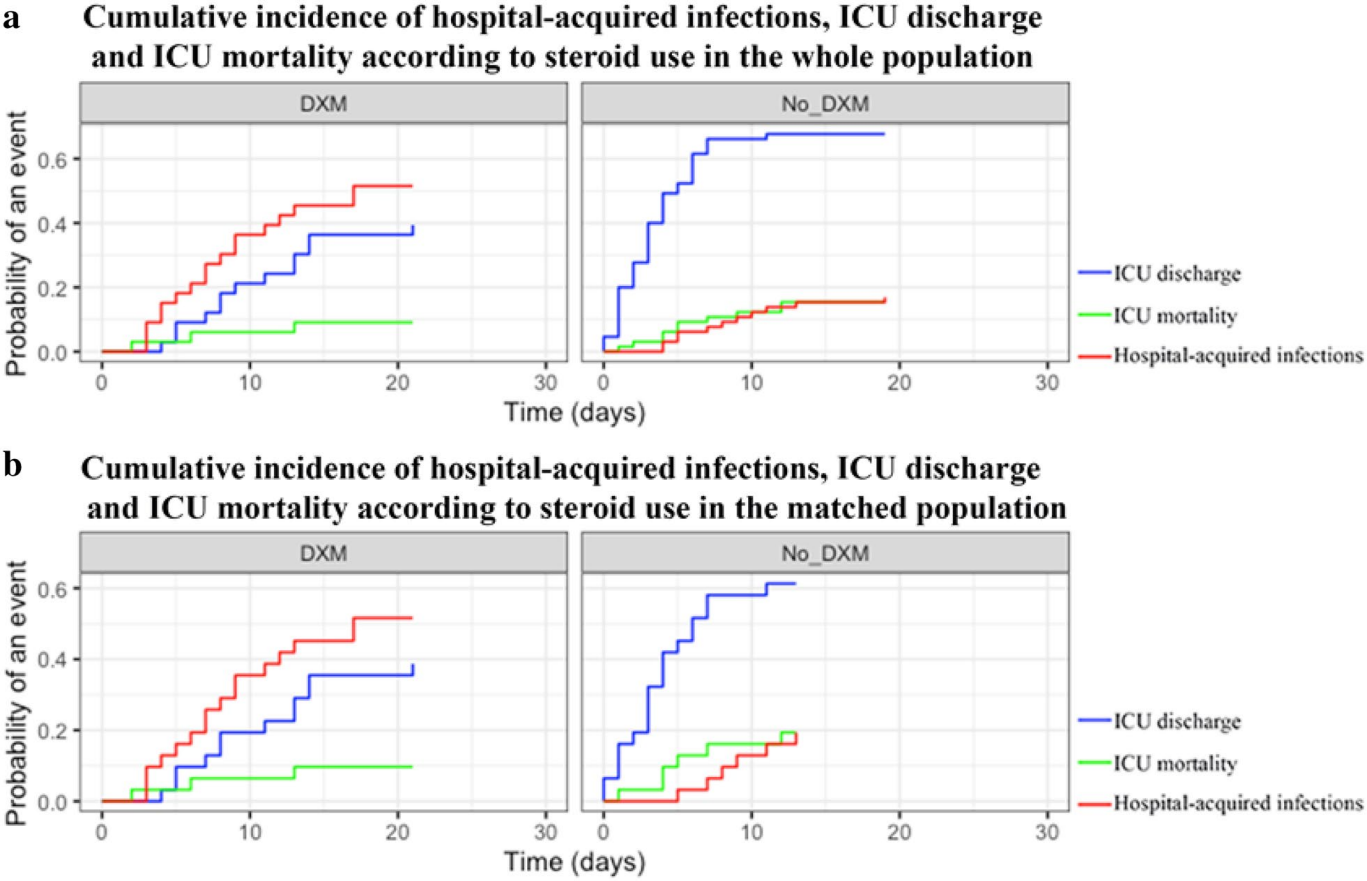
Competing risk of cumulative incidence of hospital-acquired infections in patients admitted to the ICU for severe COVID-19 according to dexamethasone intake. **a** Competing risk of hospital-acquired infections (red), ICU mortality (green) and ICU discharge (blue) in patients according to dexamethasone intake before matching. **b** Competing risk of hospital-acquired infections (red), ICU mortality (green) and ICU discharge (blue) in patients according to dexamethasone intake after matching on dexamethasone using a propensity score analysis

Cumulative incidence of hospital-acquired infections in patients treated with dexamethasone was of 36% [20–53] at 10 days compared to 12% [4–20] at 10 days for those who did not receive dexamethasone (*p* < 0.001; Fig. 4a). Dexamethasone was significantly associated with cumulative incidence of hospital-acquired infections when analyzed in the subset of patients requiring invasive mechanical ventilation (cumulative incidence of 26% [8–46] at 10 days compared to 11% [2–20]; *p* = 0.004).

After matching on variables associated with the use of dexamethasone (Additional file 1: Tables S3, S4; Additional file 2: Fig. S1), cumulative incidence of hospital-acquired infections remained higher in patients receiving dexamethasone (35% [18–52] vs. 13% [1–25] at 10 days, respectively, *p* = 0.03; Fig. 4b). Dexamethasone was no longer associated with cumulative incidence of hospital-acquired infections in the subset of patients requiring mechanical ventilation (cumulative incidence of 44% [24–63] at 10 days compared to 23% [2–44], *p* = 0.16). Dexamethasone treatment had no influence on the survival rate at day-28 (Additional file 2: Fig. S2).

## Discussion

This study focuses on concomitant infections during severe COVID-19. Our data suggest that these patients have a high incidence density of hospital-acquired infections (167 [100–225] events per 1000 days of ICU exposure) including VAP (6 [2–8] events per 1000 h of mechanical ventilation). Infection rate was particularly high in mechanically ventilated patients, with a cumulative incidence of 40%. Infections predominantly involved hospital-acquired bacteria with Gram-negative bacilli. Dexamethasone was associated with hospital-acquired infections. After matching, dexamethasone remained associated with hospital-acquired infection, but this effect was no longer detected in ventilated patients.

Our results are in line with previous reports. A descriptive study reported the occurrence *Mycoplasma pneumonia* infection in 8.6% in 140 COVID-19 patients in Wuhan [20]. Previous reports also underlined the risk of hospital-acquired infections [3] including plurimicrobial infections with *Acinetobacter baumannii*, *Klebsiella pneumoniae* and *Aspergillus flavus* in a patient [21], and the rate of acquired pneumonia of nearly 20% in ICU patients [22, 23]. As in our study, high frequency of Gram-negative bacilli was noted [12, 22, 24]. Our results are consistent with a recently published multicenter cohort reporting a 50% VAP incidence at day 28 [12]. This incidence was confirmed to be higher than in patients requiring mechanical ventilation for influenza infection or other etiologies [12–14]. Nevertheless, incidence density of nosocomial infection is higher than in previous studies, suggesting the high number of immunocompromised patients may have favored nosocomial infection [14]. Interestingly, nosocomial infection rate does not seem to be influenced by preexisting antibiotic therapies, infection incidence occurring in 33% of patients with previous antibiotic therapy when compared to 38% in patients without.

Non-bacterial infections were uncommon. In line with previous studies [21], fungal infections were uncommon. Two candidemia were identified with an overall incidence close to the one found in the general ICU setting [25]. Similarly, no patient had concomitant influenza or influenza-like community-acquired infections. This point is, however, consistent with the timing of COVID-19 in France, which occurred after the flu epidemic.

Several factors may explain the observed high incidence of infection in this study. First, in line with the high rate of patients with solid tumors or hematological malignancy in our institution, rate of immunocompromised patients was high in this cohort, when compared to a 4% incidence in a recent multicenter cohort of critically ill COVID patients [26]. In addition, patients infected with SARS-CoV-2 are prone to secondary infections, due to their severity, ICU length of stay, and sepsis-induced immunoparalysis. Lymphopenia was reported as a common feature in patients with COVID-19 and a critical factor associated with the severity and mortality [22, 10]. Lymphopenia was shown to affect both peripheral CD4 and CD8 T lymphocytes, considerably reducing their absolute number [27] while their condition was rather hyperactivated, as evidenced by the increase in the expression of HLA-DR [10]. Another interesting feature is the association between cytokine levels in COVID-19 with the severity of the clinical picture [24]. In our study, neither IL-6 dosage nor lymphopenia were associated with the risk of hospital-acquired infections.

Our study has several limitations. First, the retrospective and single-centered design may influence external validity of our findings, and deserves to be taken into account. The decision to initiate dexamethasone was made on consensus among attending physicians based on hospital length of stay and therefore with risk exposure to hospital-acquired infections. However, dexamethasone was no longer associated to acquired infections after matching on the risk to receive this treatment in the subset of patients requiring mechanical ventilation. This result suggests that dexamethasone effect was potentially confounded by severity of illness and need for mechanical ventilation. Consequently, this study cannot tease out an independent effect of dexamethasone. In this line, a high rate of immunocompromised patients may lead to an overestimation of nosocomial infection rate in COVID-19 patients. Although this limit may deserve to be kept in mind while interpreting our findings, our results are in line with findings of recent studies in this field [12–14]. In addition, the study is relatively small and may lack power to discriminate effect of dexamethasone after matching in ventilated patients. Last, in some subgroups, the limited number of available patients resulted in limited statistical power.

In conclusion, this study suggests a high incidence of infection in patients with severe COVID-19. The rate of bacterial community-acquired infections at ICU admission reaches 7%, which does not support systematic use of empirical antibiotic therapy at ICU admission. Antibiotic therapy should however be discussed for most severe patients and rapid de-escalation discussed in absence of patent bacterial infection. Futures studies are needed to further explore the influence of lower steroid dose (such as those used in recent RCTs) on nosocomial infection rate.

## Supplementary Information

**Additional file 1**:** Table S1. **Characteristics of all patients admitted to the ICU for severe COVID-19.** Table S2. **Patient’s characteristics according to ICU outcome.** Table S3. **Patient’s characteristics according to dexamethasone treatment.** Table S4. **Patient’s characteristics according to dexamethasone treatment after matching**.**

**Additional file 2:** **Figure S1**. **A** Propensity score distribution before (gray) and after (black) matching according to treatment with dexamethasone of severe COVID-19 patients. Covariates included in the model were cardiac diseases, delay of first symptoms to admission, and eculizumab. **B** Standardized mean difference before and after matching across the main variables of interest. **Figure S2**. Cumulative survival of patients admitted to the ICU for severe COVID-19 according to the administration of dexamethasone. Dexamethasone (red) and no dexamethasone (blue) survival curves are obtained by Kaplan Meier analysis and compared using Log Rank test. Covariate included in the model were cardiac disease, delay from symptoms onset to admission, mechanical ventilation and eculizumab.

## Data Availability

The datasets used and/or analyzed during the current study are available from the corresponding author on reasonable request.

## Abbreviations

ARBs: Angiotensin receptor blockers
BMI: Body mass index
ICU: Intensive care unit
COPD: Chronic obstructive pulmonary disease
CPK: Creatine phosphokinase
NSAIDs: Non-steroid anti-inflammatory drugs
SAPSII: Simplified Acute Physiology Score
SARS-CoV-2: Severe acute respiratory syndrome CoronaVirus 2
SOT: Solid organ transplantation
VAP: Ventilator-associated pneumonia
