# Supplementary material for: Infectious events in patients with severe COVID-19: results of a cohort of patients with high prevalence of underlying immune defect
Source: Ann Intensive Care. 2021 May 25;11:83. doi: 10.1186/s13613-021-00873-x (PMC8148396; doi:10.1186/s13613-021-00873-x)
Supplement: Supplementary file 1 — Additional file 1: Table S1. Characteristics of all patients admitted to the ICU for severe COVID-19. Table S2. Patient’s characteristics according to ICU outcome. Table S3. Patient’s characteristics according to dexamethasone treatment. Table S4. Patient’s characteristics according to dexamethasone treatment after matching. [file 13613_2021_873_MOESM1_ESM.docx]

**Infectious events in patients with severe COVID-19: Results of a cohort of patients with high prevalence of underlying immune defect**

Anastasia Saade *et al.*

*Additional file 1: appendix*

**Table S1.** Characteristics of all patients admitted to the ICU for severe COVID-19.

|  | | **Overall (n=100)** | |
| --- | --- | --- | --- |
| **Age (years)** | | 59 [53-67] | |
| **Male** | | 73 (73) | |
| **Comorbidities** | |  | |
| BMI (kg/m^2^) | | 28 [24-31] | |
| Cardiac disease | | 18 (18) | |
| COPD | | 2 (2) | |
| Diabetes, | | 27 (27) | |
| Chronic kidney disease | | 14 (14) | |
| Hypertension | | 50 (50) | |
| Solid tumor | | 9 (9) | |
| Hematological malignancy | | 15 (15) | |
| SOT | | 10 (10) | |
| **Any ARBs** | | 27 (27) | |
| **Delay since onset (days)** | | 8 [5-12] | |
| **Exposure before admission** | |  | |
| NSAIDs | | 1 (1) | |
| Corticosteroids | | 19 (19) | |
| Antibiotics | | 45 (45) | |
| **Hospital stay before ICU (days)** | | 1 [0-2] | |
| **ICU admission** | |  | |
| SAPSII | | 26 [18-39] | |
| O2 (L/min) | | 8 [5-12] | |
| Focal opacity at Chest Xray | | 5 (5) | |
| PaO_2_/FiO_2_ | | 152 [111-227] | |
| Leukocytes (G/L) | | 7 [5-9] | |
| Lymphocytes (G/L) | | 0.79 [0.52-1.11] | |
| Lactate (mmol/L) | | 1.20 [0.90-1.50] | |
| CPK (UI/L) | | 167 [79-371] | |
| Creatinine (μmol/L) | | 80 [64-120] | |
| IL-6 (ng/mL) | | 82 [47-129] | |
| Gammaglobulin (g/L) | | 9 [7-12] | |
| **Therapeutics** | |  | |
| Dexamethasone | | 33 (33) | |
| Eculizumab | | 10 (10) | |
| Tocilizumab | | 5 (5) | |
| Remdesivir | | 1 (1) | |
| Hydroxychloroquine | | 6 (6) | |
| Lopinavir | | 13 (13) | |
| Mechanical ventilation | | 54 (54) | |
| Vasopressors | | 48 (49) | |
| Renal replacement therapy | | 12 (12) | |
| **Infectious event** | | 36 (36) | |
| **Community-acquired infections** | | 7 (7) | |
| **Hospital-acquired infections** | |  | |
| Early | | 14 (14) | |
| Late | | 15 (15) | |
| **VAP (/1000 hours of MV)** | | 0.0 [0.0-1.3] | |
| **ICU mortality** | | 28 (28) | |
| **ICU length of stay (days)** | | 6 [3-13] | |

Data are reported as absolute value with percentage for categorical variables or median with interquartile interval for quantitative variables.

ARBs: angiotensin receptor blockers; BMI: Body mass index; COPD: Chronic Obstructive Pulmonary Disease; CPK: Creatin PhosphoKinase; ICU: Intensive Care Unit; MV: Mechanical Ventilation; NSAIDs: non-steroid anti-inflammatory; SAPSII: Simplified Acute Physiology Score II; SOT: Solid Organ Transplantation; VAP: Ventilator-Associated Pneumonia.

**Table S2.** Patient’s characteristics according to ICU outcome.

|  | **Alive (n=72)** | **Deceased (n=28)** | ***p*** |
| --- | --- | --- | --- |
| **Age (years)** | 57 [50-64] | 67 [58-70] | <0.001 |
| **Male** | 51 (71) | 22 (79) | 0.595 |
| **Comorbidities** |  |  |  |
| BMI (kg/m^2^) | 28 [24-32] | 28 [25-29] | 0.36 |
| Cardiac disease | 9 (13) | 9 (32) | 0.05 |
| COPD | 0 (0) | 2 (7) | 0.14 |
| Diabetes, | 16 (22) | 11 (39) | 0.14 |
| Chronic kidney disease | 8 (11) | 6 (21) | 0.31 |
| Hypertension | 33 (46) | 17 (61) | 0.27 |
| Solid tumor | 4 (6) | 5 (18) | 0.12 |
| Hematological malignancy | 8 (11) | 7 (25) | 0.15 |
| SOT | 7 (10) | 3 (11) | 1.00 |
| **Any ARBs** | 19 (27) | 8 (30) | 0.23 |
| **Delay since onset (days)** | 9 [6-13] | 8 [4-10] | 0.21 |
| **Exposure before admission** |  |  |  |
| NSAIDs | 1 (1) | 0 (0) | 1.00 |
| Steroids | 11 (15) | 8 (29) | 0.22 |
| Antibiotics | 34 (47) | 11 (40) | 0.62 |
| **ICU admission** |  |  |  |
| SAPSII | 22 [18-32] | 42 [33-56] | <0.001 |
| O2 (L/min) | 9 [6-12] | 7 [2-15] | 0.65 |
| Focal opacity at Chest Xray | 2 (3) | 3 (11) | 0.26 |
| PaO_2_/FiO_2_ | 165 [115-254] | 124 [88-159] | 0.02 |
| Leukocytes (G/L) | 7 [5-9] | 7 [5-9] | 0.68 |
| Lymphocytes (G/L) | 0.83 [0.56-1.26] | 0.71 [0.51-0.88] | 0.08 |
| Lactate (mmol/L) | 1.2 [0.9-1.6] | 1.2 [0.8-1.4] | 0.90 |
| CPK (UI/L) | 156 [77-369] | 169 [84-405] | 0.56 |
| Creatinine (μmol/L) | 74 [61-99] | 105 [75-176] | 0.01 |
| IL-6 (ng/mL) | 79 [39-112] | 116 [81-269] | 0.00 |
| Gammaglobulin (g/L) | 9 [7-12] | 9 [7-12] | 0.90 |
| **Therapeutics** |  |  |  |
| Dexamethasone | 21 (29) | 12 (44) | 0.23 |
| Eculizumab | 6 (8) | 4 (14) | 0.60 |
| Tocilizumab | 5 (7) | 0 (0) | 0.36 |
| Mechanical ventilation | 27 (38) | 27 (96) | <0.001 |
| Vasopressors | 25 (35) | 23 (85) | <0.001 |
| Renal replacement therapy | 4 (6) | 8 (30) | 0.003 |
| **Infectious events** | 19 (26) | 18 (64) | 0.001 |
| **VAP (/1000 hours of MV)** | 0.0 [0.0-0.0] | 0.0 [0.0-7.81] | 0.020 |

Data are reported as absolute value with percentage for categorical variables or median with interquartile interval for quantitative variables.

ARBs: angiotensin receptor blockers; BMI: Body mass index; COPD: Chronic Obstructive Pulmonary Disease; CPK: Creatin PhosphoKinase; ICU: Intensive Care Unit; MV: Mechanical Ventilation; NSAIDs: non-steroid anti-inflammatory; SAPSII: Simplified Acute Physiology Score II; SOT: Solid Organ Transplantation; VAP: Ventilator-Associated Pneumonia.

**Table S3.** Patient’s characteristics according to dexamethasone treatment.

|  | **No dexamethasone (n=66)** | **Dexamethasone* (n=33)** | ***p*** |
| --- | --- | --- | --- |
| **Age (years)** | 58 [52-67] | 59 [54-67] | 0.79 |
| **Male** | 47 (71) | 25 (76) | 0.81 |
| **Comorbidities** |  |  |  |
| BMI (kg/m^2^) | 28 [24-32] | 28 [24-31] | 0.60 |
| Cardiac disease | 15 (23) | 3 (9) | 0.17 |
| COPD | 2 (3) | 0 (0) | 0.80 |
| Diabetes | 15 (23) | 12 (36) | 0.23 |
| Chronic kidney disease | 9 (14) | 5 (15) | 1.00 |
| Hypertension | 32 (49) | 17 (52) | 0.94 |
| Solid tumor | 7 (11) | 2 (6) | 0.71 |
| Hematological malignancy | 11 (17) | 3 (9) | 0.48 |
| SOT | 6 (9) | 4 (12) | 0.91 |
| **Any ARBs** | 16 (24) | 11 (33) | 0.21 |
| **Delay since onset (days)** | 7 [5-10] | 10 [8-14] | 0.00 |
| **Exposure before admission** |  |  |  |
| NSAIDs | 1 (2) | 0 (0) | 1.00 |
| Corticosteroids | 11 (17) | 8 (24) | 0.53 |
| Antibiotics | 27 (41) | 18 (55) | 0.28 |
| **ICU admission** |  |  |  |
| SAPSII | 26 [18-39] | 28 [21-38] | 0.86 |
| O2 (L/min) | 7 [4, 12] | 9 [6, 15] | 0.09 |
| Focal opacity at Chest Xray | 3 (5) | 2 (6) | 1.00 |
| PaO_2_/FiO_2_ | 153 [112-229] | 150 [111-224] | 0.75 |
| Leukocytes (G/L) | 7 [5-9] | 7 [5-9] | 0.98 |
| Lymphocytes (G/L) | 0.77 [0.49-1.07] | 0.86 [0.57-1.27] | 0.39 |
| Lactate (mmol/L) | 1.20 [0.80-1.50] | 1.20 [1.00-1.40] | 0.83 |
| CPK (UI/L) | 170 [76-372] | 145 [83-355] | 0.93 |
| Creatinine (μmol/L) | 81 [66-135] | 75 [58-100] | 0.31 |
| IL-6 (ng/mL) | 82 [39-126] | 91 [64-136] | 0.23 |
| Gammaglobulin (g/L) | 9 [7-11] | 10 [7-12] | 0.60 |
| **Therapeutics** |  |  |  |
| Eculizumab | 2 (3) | 8 (24) | 0.00 |
| Tocilizumab | 5 (8) | 0 (0) | 0.26 |
| Mechanical ventilation | 27 (41) | 26 (79) | 0.00 |
| Vasopressors | 26 (39) | 22 (67) | 0.02 |
| Renal replacement therapy | 6 (9) | 6 (18) | 0.33 |
| **Infectious events** | 15 (23) | 20 (61) | <0.001 |
| **Hospital-acquired infections** |  |  |  |
| Early | 5 (8) | 9 (27) |  |
| Late | 7 (11) | 8 (24) |  |
| **VAP (/1000 hours of MV)** | 0.0 [0.0-0.0] | 0.0 [0.0-6.9] | 0.003 |
| **ICU mortality** | 15 (23%) | 12 (36%) | 0.23 |
|  | | | |

*Delay between dexamethasone and nosocomial infection is of 3 [IQR 2-4 days].

Data are reported as absolute value with percentage for categorical variables or median with interquartile interval for quantitative variables.

ARBs: angiotensin receptor blockers; BMI: Body mass index; COPD: Chronic Obstructive Pulmonary Disease; CPK: Creatin PhosphoKinase; ICU: Intensive Care Unit; MV: Mechanical Ventilation; NSAIDs: non-steroid anti-inflammatory; SAPSII: Simplified Acute Physiology Score II; SOT: Solid Organ Transplantation; VAP: Ventilator-Associated Pneumonia.

**Table S4.** Patient’s characteristics according to dexamethasone treatment after matching.

|  | | **No dexamethasone (n=31)** | **Dexamethasone (n=31)** | ***p*** |
| --- | --- | --- | --- | --- |
| **Age (years)** | | 57 [49-64] | 59 [54-67] | 0.50 |
| **Male** | | 20 (64) | 24 (77) | 0.40 |
| **Comorbidities** | |  |  |  |
| Cardiac disease | | 1 (3) | 2 (7) | 1.00 |
| COPD | | 1 (3) | 0 (0) | 1.00 |
| Diabetes | | 4 (13) | 10 (32) | 0.13 |
| Chronic kidney disease | | 3 (10) | 4 (13) | 1.00 |
| Hypertension | | 14 (45) | 15 (48) | 1.00 |
| SOT | | 1 (3) | 3 (10) | 0.61 |
| **Delay since onset (days)** | | 9 [7-13] | 10 [8-14] | 0.34 |
| **ICU admission** | |  |  |  |
| O2 (L/min) | | 9 [6-12] | 9 [6-15] | 0.76 |
| Focal opacity at Chest Xray | | 2 (7) | 2 (7) | 1.00 |
| PaO_2_/FiO_2_ | | 151 [110-229] | 152 [114-225] | 0.83 |
| Leukocytes G/L | | 7 [6-9] | 7 [5-9] | 0.12 |
| IL-6 ng/mL | | 82 [50-173] | 94 [63-143] | 0.65 |
| **Therapeutics** | |  |  |  |
| Eculizumab | | 2 (7) | 7 (23) | 0.15 |
| Mechanical ventilation | | 17 (55) | 25 (81) | 0.06 |
| Vasopressors | | 16 (52) | 21 (67) | 0.30 |
| Renal replacement therapy | | 4 (13) | 5 (16) | 1.00 |
| **Hospital-acquired infections** | | 4 (13) | 12 (39) | 0.04 |
| **VAP (/1000 hours of MV)** | | 0 [0-0] | 0 [0-6] | 0.031 |
| **ICU mortality** | | 15 (23%) | 12 (36%) | 0.40 |
|  |  |  |  |  |

Data are reported as absolute value with percentage for categorical variables or median with interquartile interval for quantitative variables.

ARBs: angiotensin receptor blockers; COPD: Chronic Obstructive Pulmonary Disease; ICU: Intensive Care Unit; MV: Mechanical Ventilation; SOT: Solid Organ Transplantation.

**Figure S1. A)** Propensity score distribution before (gray) and after (black) matching according to treatment with dexamethasone of severe COVID-19 patients. Covariates included in the model were cardiac diseases, delay of first symptoms to admission, and eculizumab. **B)** Standardized mean difference before and after matching across the main variables of interest.

**Figure S2.** Cumulative survival of patients admitted to the ICU for severe COVID-19 according to the administration of dexamethasone.

Dexamethasone (red) and no dexamethasone (blue) survival curves are obtained by Kaplan Meier analysis and compared using Log Rank test.

Covariate included in the model were cardiac disease, delay from symptoms onset to admission, mechanical ventilation and eculizumab.
