# Supplementary figures and images for: Infectious events in patients with severe COVID-19: results of a cohort of patients with high prevalence of underlying immune defect
Source: Ann Intensive Care. 2021 May 25;11:83. doi: 10.1186/s13613-021-00873-x (PMC8148396; doi:10.1186/s13613-021-00873-x)

## Slide 1
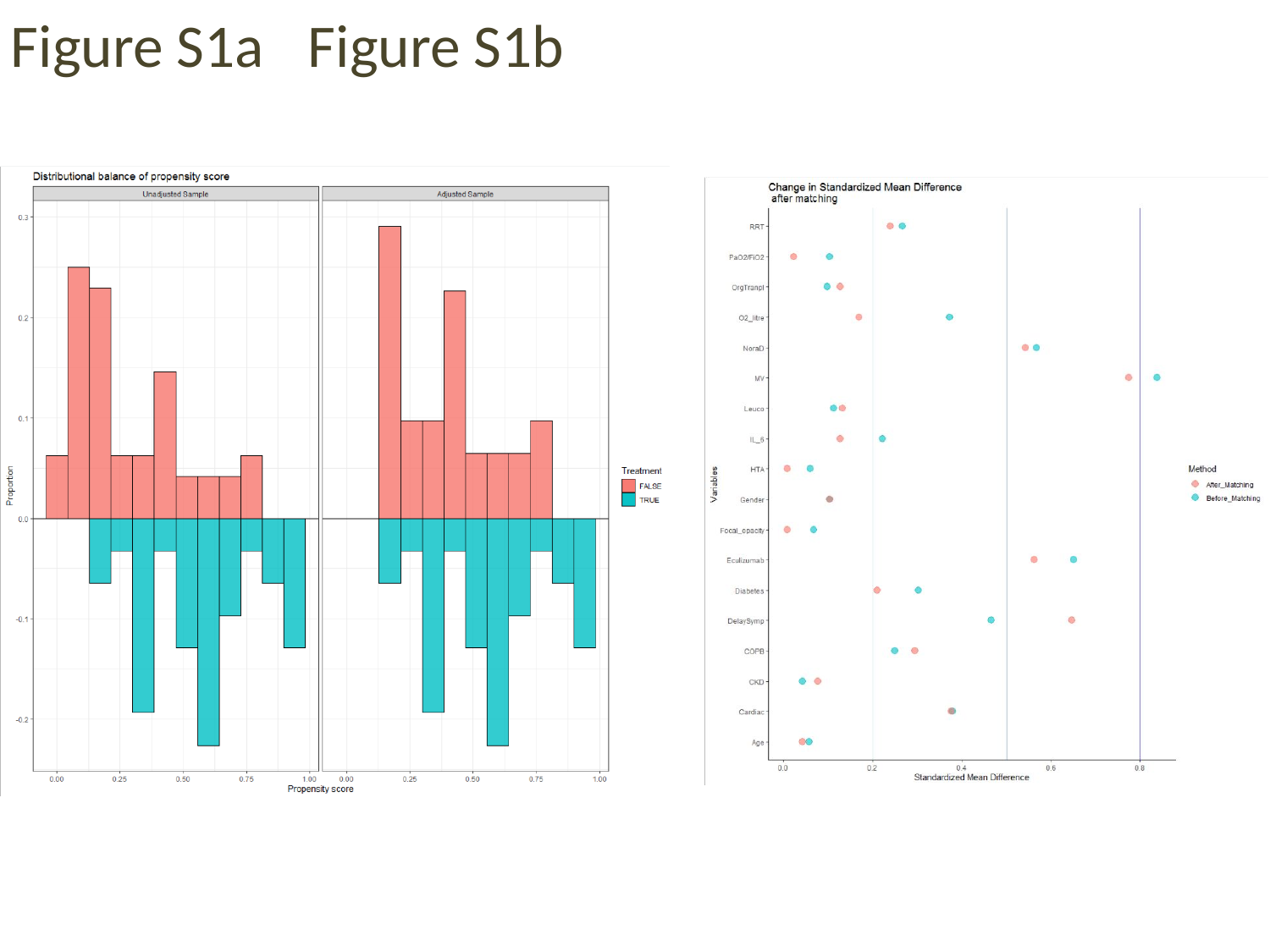

# Figure S1a			Figure S1b

## Slide 2
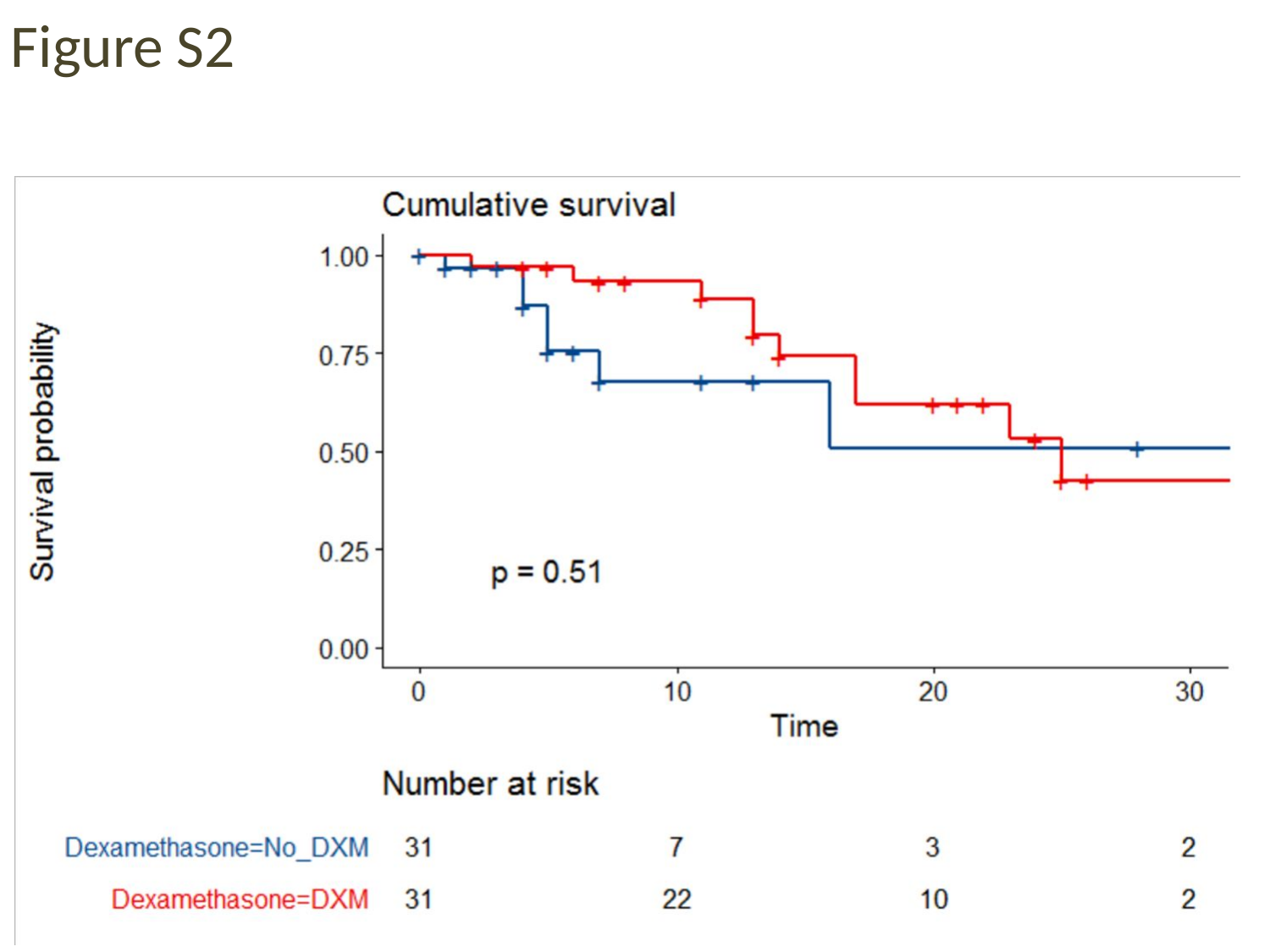

# Figure S2

Supplement: Supplementary file 2 — Additional file 2: Figure S1. A Propensity score distribution before (gray) and after (black) matching according to treatment with dexamethasone of severe COVID-19 patients. Covariates included in the model were cardiac diseases, delay of first symptoms to admission, and eculizumab. B Standardized mean difference before and after matching across the main variables of interest. Figure S2. Cumulative survival of patients admitted to the ICU for severe COVID-19 according to the administration of dexamethasone. Dexamethasone (red) and no dexamethasone (blue) survival curves are obtained by Kaplan Meier analysis and compared using Log Rank test. Covariate included in the model were cardiac disease, delay from symptoms onset to admission, mechanical ventilation and eculizumab. [file 13613_2021_873_MOESM2_ESM.pptx]
